# Nitrogen hurdle of host alternation for a polyphagous aphid and the associated changes of endosymbionts

**DOI:** 10.1038/srep24781

**Published:** 2016-04-20

**Authors:** Yan-Hong Liu, Zhi-Wei Kang, Ya Guo, Guo-Shuai Zhu, M. Mostafizur Rahman Shah, Yue Song, Yong-Liang Fan, Xiangfeng Jing, Tong-Xian Liu

**Affiliations:** 1 State Key Laboratory of Crop Stress Biology for Arid Areas, and Key Laboratory of Integrated Pest Management on the Loess Plateau of Ministry of Agriculture, Northwest A&F University, Yangling, Shaanxi, 712100 China

## Abstract

Low proportion of essential amino acids (EAAs) is one of the barriers for animals to use phloem as a diet. Endosymbionts with EAAs synthesis functions are considered crucial for ameliorating the lack of EAAs in insects’ diets. In this study, we transferred the insects from a cabbage-reared *Myzus persicae* population onto 3 new plant species including eggplant, tobacco and spinach. The performance on these plants was evaluated and the dynamics of endosymbionts in relation to this host alternation were recorded. We found that the EAAs ratio in phloem was largely determined by the concentrations of non-essential amino acids and the higher proportion of EAAs seemed to favor the population establishment on new plant species and the growth of primary endosymbionts inside insects, which indicated that nitrogen quality was an important factor for aphids to infest and spread on new plant hosts.

Insect-plant interactions have long been one of core research areas among entomologists, especially considering its crucial importance from an applied point of view. How insect herbivores infest and spread on hosts is undoubtedly an intriguing aspect of these interactions. Nitrogen nutrients, especially essential amino acids (EAAs), in hosts are critical in determining if insects can establish on hosts successfully^1^,^2^,^3^,^4^. Phloem sap generally contains low ratio of EAAs but symbiotic microorganisms with reduced genomes biased in EAAs and vitamins syntheses enable phloem-feeders overcome this barrier^5^,^6^,^7^,^8^.

Amino acid composition in phloem varies among different plant species^9^. How phloem-feeders response to the nutritional changes among different hosts, and the related dynamics of endosymbionts are interesting questions. Artificial diet is often used to study the effect of nutrients including amino acids on herbivorous insects because of its simplicity by excluding the interference of non-target chemicals especially allelochemicals commonly found in plants. Phloem is generally free of defensive chemicals, which is equivalent to the first property of artificial diets, and two major chemical groups commonly found in phloem are sugars, in excess of what phloem-feeders usually need, and amino acids, more weighted in non-essential ones^5^. At the same time, as an advantage over artificial diet, phloem sap can directly reflect the real conditions insects meet in nature. For example, the turgor pressure (0.2–1 MPa) in phloem which can affect ingestion behavior of aphids is one such factor^10^.

The green peach aphid, *Myzus persicae* (Sulzer) (Hemiptera: Aphididae), is an important pest feeding on more than 400 plant species in over 50 families around the world^11^. It causes enormous losses to crop yields by sucking plant sap and by transmitting viral diseases^11^,^12^. Like other phloem-feeders, *M. persicae* also bears endosymbionts in the genus of *Buchnera*, which can synthesize EAAs for the hosts^13^,^14^. In this study, we examined the amino acid composition in the phloem of different plant species and then evaluated its potential effects on aphid’s performance. *M. persicae*, and 4 different plant species in 3 families which have been recorded as its hosts were used. The endosymbionts in these insects were identified and quantified, and the dynamics of these bacteria in response to the change of amino acid composition were also recorded. As far as we know, this is the first attempt to tackle the interactions among plants, aphids and endosymbionts in aphids from nitrogen nutritional aspect.

## Results

### Performance of cabbage-reared *M. persicae* after transferred to non-native host plant species

**Figure 1:**
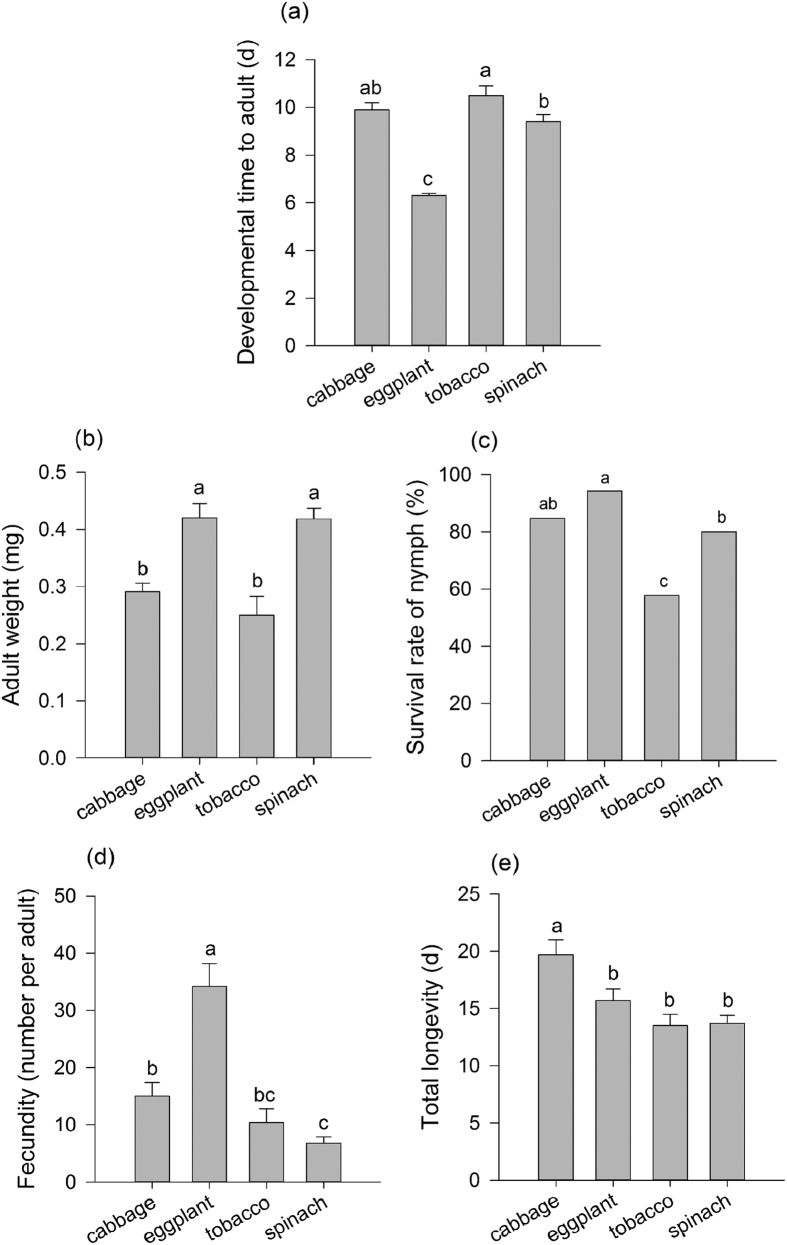
Performance of cabbage-reared *Myzus persicae* on different plant species. (**a**) Developmental time (days for aphids to reach the adult stage). (**b**) Survival rate (percentage of aphids survived to the adult stage). (**c**) Adult weight (mg). (**d**) Fecundity (the number of offspring laid per adult). (**e**) Total longevity (total number of days aphids lived) Different letters above the bars indicate significant difference among different treatments (*P* < 0.05).

Overall, cabbage-reared aphids can establish on all three new plant species, i.e., eggplant, tobacco and spinach, but the performance was different. Interestingly, the performance was better on one of the new plant species, i.e., eggplant, than on cabbage (Fig. 1). Extraordinarily, in contrast to those on cabbage, the larval developmental time on eggplant was about 4 days less (*P* < 0.001) but the insects gained 33% more weight (*F*_3,131_ = 13.369, *P* < 0.001) (Fig. 1a,b). Aphid adults on spinach were also significantly heavier than those on cabbage in spite of a similar developmental time. No differences in developmental time and adult weight were found between those reared on tobacco and cabbage but the survival rate was significantly lower on tobacco than on cabbage (Fig. 1c). In contrast, the survival rate of aphids on eggplant was significantly higher than that on spinach (*P* = 0.03), both of which were similar to that on cabbage. Fecundity was the highest on eggplant and the lowest on spinach although the adults weighed similarly between eggplant and spinach (Fig. 1b,d). Total longevity of aphids on eggplant, tobacco and spinach was similar and significantly lower than that on cabbage (Fig. 1e).

### Amino acids in plant phloem sap

**Figure 2:**
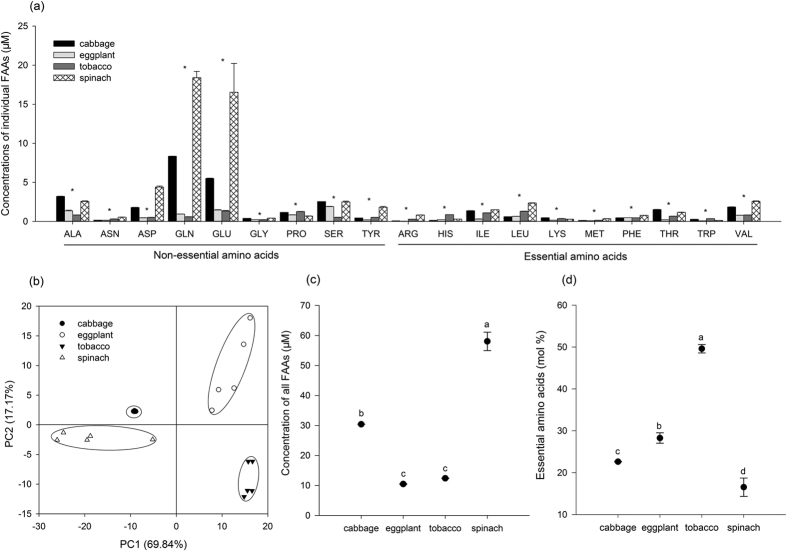
Concentrations of individual amino acid and total free amino acids, the proportion of essential amino acids and amino acid composition profiles in the four plant phloem. (**a**) Concentrations of individual free amino acid (μM). (**b**) Amino acid composition profiles (PCA). (**c**) Concentration of total free amino acids (μM). (**d**) Proportion of essential amino acids (mol%). Asterisk or different letters above the error bars indicate significant differences among different treatments (*P* < 0.05, Tukey’s HSD test).

All protein amino acids were detected in phloem exudate samples of cabbage, eggplant, tobacco and spinach leaves (Fig. 2a). Cysteine was detected but not included in the analysis because the standard cannot dissolve in water.

Concentrations of individual free amino acid detected among the four plant species varied significantly (Fig. 2a). Except for leucine in tobacco, the 3 most abundant amino acids were all non-essential amino acids in each plant species: glutamine (27.4%), glutamic acid (18.1%) and alanine (10.5%) in cabbage; serine (20.2%), glutamic acid (13.1%) and alanine (12.2%) in eggplant; glutamic acid (11.0%), leucine (10.3%) and proline (10.0%) in tobacco; glutamine (33.8%), glutamic acid (23.7%) and aspartic acid (7.1%) in spinach. A principle component analysis (PCA) with 87.01% variance of the data indicated that the variation of amino acids within sample replicates appeared to be smaller than that among different plant species (Fig. 2b). Eggplant and tobacco contained the lowest total amino acids (TAAs), and spinach had the highest TAAs (Fig. 2c, *F*_3,16_ = 207.14, *P* < 0.001). The average contribution of EAAs to TAAs varied significantly among the four plant species, ranging from 17.8 mol % in spinach to 51.1 mol % in tobacco (Fig. 2d, *F*_3,16_ = 788.50, *P* < 0.001).

### Endosymbionts in *M. persicae*

*B. aphidicola* (GenBank accession number: KM577346) and only one secondary symbiont, *S. symbiotica* (GenBank accession number: KM577348), were identified by diagnostic PCR. *S. symbiotica* (GenBank accession number: KM577347) was also identified by universal primers PCR. A phylogenetic analysis indicated that *S. symbiotica* harboured by the experimental aphids belonged to cluster A classified in a previous study and this cluster also included the endosymbiont from *Acyrthosiphon pisum*^15^ (Fig. S1). Additionally, this secondary symbiont was detected in all aphids examined 10 days after they were transferred to the different plant species (Table 1).

The relative abundance of *Buchnera* cells differed significantly (*F*_3,29_ = 26.66, *P* < 0.001) in aphids when they were fed on different plant species (Table 2). *Myzus*-tobacco and *Myzus*-eggplant had similar relative abundance and both had significantly more primary symbionts than *Myzus*-cabbage. In contrast, the relative density of *Buchnera* cells in *Myzus*-spinach was significantly lower than that in *Myzus*-cabbage.

The relative abundance of *S. symbiotica* was much lower than *Buchnera* and was significantly different (*F*_3,22_ = 12.92, *P* < 0.001) among the aphids on the four plant species (Table 2). Similar to *Buchnear* cells, the relative amount of secondary symbiont was not different between *Myzus*-tobacco and *Myzus*-eggplant, and they were both significantly higher than that in *Myzus*-cabbage. No difference was found for the relative amount of *S. symbiotica* between *Myzus*-spinach and *Myzus*-cabbage.

## Discussion

Nitrogen, i.e., amino acids, in phloem sap is important for nutritional biology of aphids^16^,^17^. However, nitrogen quality, i.e., the composition of nitrogenous compounds, in phloem is generally poor because of the low proportion of EAAs. The survival of most aphid species is largely dependent on the supply of EAAs from the primary symbiont *Buchnera*^8^,^18^,^19^. Phloem collected through stylets is what aphids ingest but the quantity collected by this way is usually too low for further analysis. Thus, we measured amino acid concentrations in different plant species by EDTA-exudation, a method preventing sieve element sealing and allowing us to get enough volume to measure amino acids. It has been adopted in many studies^16^,^20^,^21^,^22^,^23^,^24^,^25^,^26^,^27^ and our results indicated that it was reliable in measuring amino acid concentrations with a relatively low variation among biological replicates (Fig. 2b).

Nitrogen quality in spinach and cabbage was apparently lower than that in the other two plant species (Fig. 2d). In contrast, EAAs concentrations in these two plant species were higher than that in the other two (*F*_3,16_ = 2270.826, *P* < 0.001; Table S1), so the low quality was caused by the high concentrations of non-essential amino acids, more specifically the high proportions of glutamine and glutamic acid (Fig. 2a,c). Other studies also showed that glutamine and glutamic acid were the two major amino acids which contributed mostly to the low nitrogen quality of the phloem of some plant species examined^16^,^18^.

Aphids performed better on the plants with higher nitrogen quality, and this trend was more obvious on eggplant than on cabbage or spinach (Figs 2d and S2 and Table S2). Considering sugars, the other major nutrients in phloem were usually in the excess supply for aphids, and the higher nitrogen quality in eggplant likely accounted for this better performance^28^. In consistence, other studies also suggest that EAAs but not non-essential amino acids limit the growth of aphids^29^,^30^. Furthermore, the significance of the nitrogen quality may be more apparent in this study than previous reports in which artificial diets were adopted because phloem turgor pressure can promote the ingestion rate to reduce the effect of diluted AAs in eggplant^31^. In contrast to this trend, the performance of the aphids on tobacco, which had the highest proportion of EAAs, was not higher than that on eggplant or even cabbage and spinach (Fig. S2 and Table S2). It has been reported that the leaf surface of tobacco was covered by high density of glandular trichomes, which could deter small sucking insects like aphids from reaching the phloem^10^. A close investigation on the leaf surface of the four plant species clearly showed that glandular trichome with the secretion on the top spread on tobacco leaf surface in high density but not on other plant leaf surfaces (Fig. S3). This special surface structure on tobacco leaves undoubtedly interfered with aphid feeding behaviour.

Although a secondary symbiont, *S. symbiotica*, was also detected in all aphids on different plant hosts, its relative abundance was much lower comparing to *Buchnera* especially considering the copy number of 16S rRNA gene in *S. symbiotica* is 7 times as that in *Buchnera*^32^,^33^. Furthermore, other studies have shown that the closely related bacterium, *S. symbiotica* in the pea aphid (Fig. S1), was not related to insect performance on the range of diets with different sugars and amino acids in the pea aphid, *A. pisum*^34^,^35^,^36^,^37^. Therefore, only the dynamic of *Buchnera* cells was discussed here.

The density of endosymbionts was higher when aphids fed on the plant species with higher nitrogen quality (Fig. S4), and this trend was interfered neither by trichomes nor by the secretion possibly because all tested aphids were the ones survived. One study also found that the relative abundance of *Buchnera* cells in the pea aphid was positively correlated with amino acid concentrations. But in that study a fixed amino acid composition was used so the effect of EAAs cannot be discriminated from the co-varied TAAs concentrations^31^. The synthesis of EAAs is achieved by a coordinated reactions mediated by the enzymes from both bacteria and insect hosts^38^. Therefore, we proposed that the population growth of *Buchnera* can be induced by the availability of nutrients in their diet.

In summary, we attempted to investigate the complex interplay among endosymbionts, the host insects, and plant hosts. Although only one clone of *M. persicae* was used and the variation of different insect populations cannot be excluded, this study shed lights on the important effects of nitrogen quality of new plant hosts on aphid infestion as well as the associated internal changes in endosymbionts. Our study indicated that *M. persicae* can successfully infest on different plant species with different amino acid profiles although the performance varied along the plants with different nitrogen qualities. Nitrogen quality is an important factor for aphids to infest on new plant hosts although others including plant defensive structure, e.g, trichomes on tobacco and turgor pressure in phloem, should also be considered^9^,^39^. The population dynamics of endosymbionts seem to be related to the supply of nutrients especially EAAs.

## Methods

### Plants and insects

Four economically important plant crops were used in this study: cabbage [*Brassica oleracea* L. (Cruciferae) var. ‘Qingan70’], eggplant [*Solanum melongena* L. (Solanaceae) var. ‘Zichangqie’], tobacco [*Nicotiana tabacum* L. (Solanaceae) var. ‘Qinyan95’], and spinach [*Spinacia oleracea* L. (Chenopodiaceae) var. ‘Diwa’]. The seeds were planted in peat moss and the seedlings 2 weeks after germination were transplanted to individual plastic pot (12 cm in diameter) and grown on potting mix of peat moss, vermiculite, and perlite at 7:1:1 ratio by volume at 25 ± 1 °C, 60 ± 5% RH and a 16L : 8D photoperiod with a light intensity of 1400–1725 lux. The plants were watered as needed and fertilized with Harvest More 20-20-20 + TE at the rate of 1 g/L water/week.

The green peach aphid *M. persicae* clone (YL-TB, green) was obtained from a single parthenogenetic female collected from a tobacco plant in a greenhouse (Key Laboratory of Applied Entomology, Northwest A&F University, Yangling, Shaanxi, China). This clone was established on cabbage for more than ten generations and the genotype was confirmed by mitochondrial COI gene (GenBank accession number: KM577343)^40^. The aphids from this population were transferred to the four different plant species (to cabbage as the control) when the plants reached about 9-leaf stage. For convenience, aphids on the four plant species were named as *Myzus*-cabbage, *Myzus*-eggplant, *Myzus*-tobacco and *Myzus*-spinach, respectively.

All experiments were conducted at 25 °C. For each individual plant of 4 different plant species, 3 clip cages were installed on 3 fully extended leaves respectively. Mixed-aged apterous adults reared on cabbage were transferred to clip cages to produce nymphs for 24 h and then removed. The nymphs were used in the following 3 experiments. Firstly, 15 10-day-old insects were collected for symbiont identification, and 30 were used to determine infection frequency. Secondly, 10 replicates of 15 10-day-old aphids were collected for the symbionts quantification by qPCR analysis. Thirdly, about 50 individual aphids on each plant species were checked twice daily to record development, fecundity, survival and adult weight until all individuals died. The weight of each adult was measured on a micro-balance (Sartorius MSA 3.6P-000-DM, Gottingen, Germany) right after the molt to adult stage.

### Amino acid analysis

Free amino acids (FAAs) in phloem sap of plant leaves were collected by the EDTA-exudation technique^16^. The third and fourth fully expanded leaves from the bottom of the five- to six-leaf stage plants were quickly cut by scalpel and the leaf petiole was immediately immersed into a 1.5 ml tube containing 1 ml of EDTA solution (5 mM, pH 7.0) and incubated in the dark for 4 h at 25 °C with high humidity RH (>90%). Leaf exudates were centrifuged at 4,500 g for 5 min at 4 °C. The ultrafiltered supernatant obtained by sterile syringe filter was stored at −70 °C until analysis.

FAAs in plants phloem were identified by their relative retention times to standards and quantified by LC-MS^41^ by comparing with authentic standards. Liquid chromatography separations were carried out with Intertsil OSD-4 C18 Column (250 mm × 3.0 mm; GL Sciences Inc., Tokyo, Japan). Amino acids were eluted by a three-step gradient: 100% mobile phase A (5% acetonitrile containing 0.1% formic acid) for 8 min, 0–100% B (100% acetonitrile containing 0.1% formic acid) linear for 2 min, 100% B for another 5 min, and 0–100% A linear for 1 min, holding the system at 100% A for 8 min with the flow rate of 0.3 ml/min. Mobile phase A was 5% acetonitrile containing 0.1% formic acid; and mobile phase B was 100% acetonitrile containing 0.1% formic acid. The elution pattern of FAAs was further confirmed by GC-MS running the identical column with the same procedure as stated above. LTQ XL^TM^ linear ion trap mass spectrometer (Thermo Scientific, Waltham, MA, USA) worked in the positive electrospray ionization (ESI) mode. Nitrogen as the sheath gas (30.0 arbitrary units) and auxiliary gas (5.0 arbitrary units). The spray voltage was set at 4.5 kV and the ion transfer capillary temperature was 275 °C. The amino acids were scanned and fragmented using data dependent MS/MS. Masses of precursor and product ions and collision energy for each amino acid were presented in Table S3. Data were acquired and processed using Xcalibur 2.1 software (Thermo Scientific, Waltham, MA, USA).

### Symbionts identification in different populations

Total DNA was extracted using a conventional sodium dodecyl sulfate (SDS) method^42^. To detect the symbionts in aphid accurately, two complementary methods were adopted. Firstly, diagnostic PCR with specific primers previously reported was used^8^. The primer sequences were listed in Table S4. Secondly, in almost all eubacteria except *Buchnera*, the 16S rRNA gene is connected to the 23S rRNA gene so universal primers that spanned these two regions were designed to detect secondary symbionts^43^, i.e., a universal forward primer in the 16S rRNA gene (‘10F’) and a reverse primer in the 23S rRNA gene (‘480R’).

After the S-symbiont identification, specific primers for the 23S rRNA gene of each specific S-symbiont were designed for fast detection of the infection frequencies (Table S4). The temperature profile for PCR was 95 °C for 3 min, 35 cycles of 95 °C for 30 s, 58 °C for 30 s, and 72 °C for 30 s, and a final extension at 72 °C for 5 min. The PCR products were sized and visualized on 1% agarose gels stained with ethidium bromide. Each discrete band was purified from the gel, and then cloned using pMD18-T vector (TaKaRa Bio Inc., Dalian, China). After verification of insert sizes, recombinant plasmids (5 recombinant plasmids for each PCR product amplified by specific primers and 50 for the PCR product by universal primers) were sequenced at Invitrogen Biological Corporation (Beijing, China).

### Phylogenetic reconstruction of symbiotic *S. symbiotica* lineages in aphids

Partial 16S rRNA gene sequences of *S. symbiotica* were obtained from a range of aphids (GenBank accession numbers see Table S5), and partial sequences of 16S rRNA gene from *E. coli* was used as the outgroup. All sequences obtained were aligned using MEGA6 and trimmed using the option of automated in TRIMAL v.1.2^44^. The neighbor-joining phylogeny tree was reconstructed by MEGA6 with default settings with 1000 bootsrapped replicates.

### Quantitative real-time PCR analysis

The relative abundance of symbionts in *Myzus*-cabbage, *Myzus*-eggplant, *Myzus*-tobacco and *Myzus*-spinach was determined by relative quantitative real-time PCR analysis (qPCR). Total DNA was extracted from 15 apterous adults for one sample using the SDS method as described above. qPCR was performed on the BIO-RAD, iQ5 multicolor Real-Time PCR machine with SYBR^®^Premix Ex Taq II (TaKaRa) and the reaction program was 95 °C for 3 min, then 40 cycles of 95 °C for 10 s, and 60 °C for 30 s. The single-copy aphid nuclear gene elongation factor 1α (EF1α) was quantified as the reference gene^45^,^46^. *Buchnera* 16S rRNA gene and *S. symbiotica* 16S rRNA gene were used as the target genes for qPCR^32^,^33^. The primer sequences for qPCR were shown in Table S4 and the specificity of the amplifications was validated by standard curves and sequencing (see Table S4). All experiments were conducted in nine to ten biological repeats and in three technical replicates for each sample and the raw data of endosymbionts abundances by qPCR were shown in Table S6. The results of the qPCR were normalized to the expression level of EF1α and calculated by 2^−Δ*C*t^ method.

### Data analysis

Raw data of developmental time, survival rate, longevity, and fecundity of *M. persicae* on different plants were analyzed by basic run of TWOSEX-MSChart, the computer program for the age-stage, two-sex life table analysis modified for parthenogenetic aphid (available at http://140.120.197.173/Ecology//Download/TWOSEX-MSChart.rar)^47^,^48^,^49^,^50^. The intrinsic rate of increase (*r*) was also obtained by using the program, TWOSEX-MSChart. Then 100,000 bootstrap replicates were used to calculate SEs of all estimates of life-table and population parameters and pair-wise comparison of these parameters with 1000 bootstrap replicates was conducted^47^,^48^,^49^,^50^.

Adult weight, endosymbiont abundance, individual amino acid concentrations, TAAs concentrations, EAAs concentrations and proportion of EAAs were checked for normal distribution using the Kolmogorov-Smirnov test before further analysis. These data were analyzed by one-way ANOVA with Tukey’s HSD test using IBM SPSS Statistics package v. 19.0 (SPSS Inc., Chicago, IL, USA). The variation among the body width of *M. persicae* adult, trichome spacing of tobacco and eggplant leaves was also analyzed by one-way ANOVA with Tukey’s HSD test using SPSS 19.0. Individual amino acid concentrations in different plant exudates were transformed to mol %, which were further analyzed via multiple-dimensional principal component analysis (PCA) using SAS Version 9.1. The variations among the amino acid composition profiles were based on first two principal components analysis. Each spot in the PCA plot represented individual sample, and distances among the spot groups showed the variation level of amino acid profiles among different treatments. A *P* < 0.05 was taken as statistical significance for all analyses.

## Additional Information

**How to cite this article**: Liu, Y.-H. *et al.* Nitrogen hurdle of host alternation for a polyphagous aphid and the associated changes of endosymbionts. *Sci. Rep.* **6**, 24781; doi: 10.1038/srep24781 (2016).

## Supplementary Material

Supplementary Information

## Figures and Tables

**Table 1:** Infection frequencies of two symbionts in *Myzus persicae* 10 days after transferring to different plant species.

Aphid	Infection frequency (%)	
	*Buchnera aphidicola*	*Serratia symbiotica*
*Myzus*-cabbage	100	100
*Myzus*-eggplant	100	100
*Myzus*-tobacco	100	100
*Myzus*-spinach	100	100

**Table 2:** The relative abundance of endosymbionts referring to *EF1α* gene in *Myzus persicae* reared on four different plants.

Aphid	Relative abundance (2^−Δ*C*t^)	
	*Buchnera aphidicola*	*Serratia symbiotica*(×10^−4^)
*Myzus*-cabbage	96.526 ± 8.011 b	0.408 ± 0.124 b
*Myzus*-eggplant	136.881 ± 10.178 a	2.608 ± 0.438 a
*Myzus*-tobacco	143.646 ± 8.737 a	3.269 ± 0.736 a
*Myzus*-spinach	61.193 ± 4.702 c	0.527 ± 0.098 b

The number of 16S rRNA gene copes in *B. aphidicola* and *S. symbiotica* is one and seven respectively. Values shown were mean ± SE. Different letters indicate significant difference of symbiont abundance among the four treatments (*P* < 0.05, Tukey’s HSD test).
