# Supplementary material for: Nitrogen hurdle of host alternation for a polyphagous aphid and the associated changes of endosymbionts
Source: Sci Rep. 2016 Apr 20;6:24781. doi: 10.1038/srep24781 (PMC4837378; doi:10.1038/srep24781)
Supplement: Supplementary Information [file srep24781-s1.pdf]

Supplementary Materials

Nitrogen hurdle of host alternation for a polyphagous aphid and the associated changes of endosymbionts

Yan-Hong Liu[#], Zhi-Wei Kang[#], Ya Guo, Guo-Shuai Zhu, M. Mostafizur Rahman Shah, Yue Song, Yong-Liang Fan, Xiangfeng Jing* and Tong-Xian Liu*

State Key Laboratory of Crop Stress Biology for Arid Areas, and Key Laboratory of Integrated Pest Management on the Loess Plateau of Ministry of Agriculture, Northwest A&F University, Yangling, Shaanxi, China 712100

# These authors contributed equally to this work

* Corresponding authors' e-mail: jxf_zb@sina.cn; txliu@nwsuaf.edu.cn, telephone: +
(86)-29-8708-2350

1. Supplementary figures

2. Supplementary tables

3. Reference cited in supplementary materials

SUPPLEMENTARY FIGURES

Figure S1 | Neighbor-joining phylogeny tree for *Serratia symbiotica* based on 16S rRNA genes. The tree was rebuilt following the instructions described previoiusly[1] and the sequence of *S. symbiotica* identified in this study was included. Symbiotic bacterial taxa were labeled as the name of the aphid species but two 16S rRNA gene sequences of *S. symbiotica* in the same aphid species were indicated by the name of the aphid species and GenBank accession numbers. The sequence of this study was highlighted with grey.

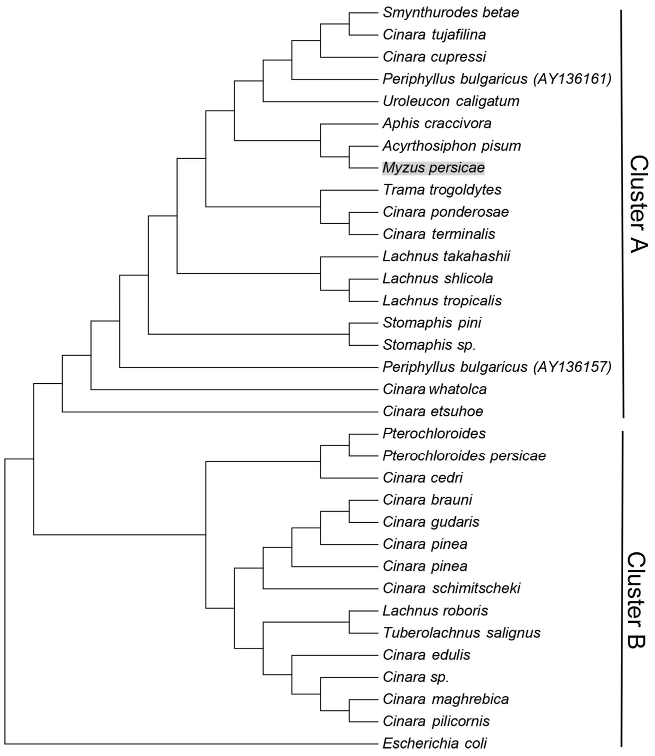

Figure S2 | Comparison between insect performance and the proportion of essential amino acids in different plant species. The intrinsic rate of increase (*r*) calculated by TWOSEX-MSChart (see method for details) was used to evaluate insect performance on different plant species. For the better illustration, both insect performance (light grey) and the proportion of essential amino acids (dark grey) were normalized to the sum of each dataset respectively (normalized intrinsic rate = the intrinsic rate on each plant species / the sum of the intrinsic rates on four plant species; normalized proportion of essential amino acids = the proportion of essential amino acids in each plant species / the sum of proportions of essential amino acids of four plant species).

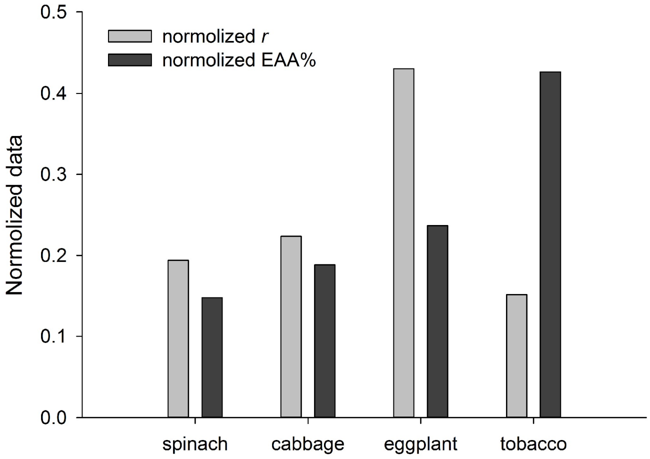

Figure S3 | Surface structures of different host plant leaves. Body width and leaf surface structure were captured using a stereo-microscope (AxioCam MRc 5 of Carl Zeiss) and calculated by the software AxioVision Rel. 4.8. Each of five 1-day-old *M. persicae* adult was measured three times. The distance between the nearest neighbor trichomes was measured with three technical replicates. Three to four pairs of trichomes were randomly selected on each of three tobacco or eggplant leaves. The leaves used were at the same stage as those used for aphid bioassay experiment. (a, b) glandular trichomes and the secretion on the surface of tobacco leaf (c) nonglandular trichomes on the surface of eggplant leaf (d) the surface of cabbage leaf (e) the surface of spinach leaf (f) body width of *M. persicae* and trichome spacing of eggplant and tobacco leaves. Different letters show significant difference among the body width and trichome spacing of two plant species ($F_{2, 22} = 39.781$, $P < 0.001$).

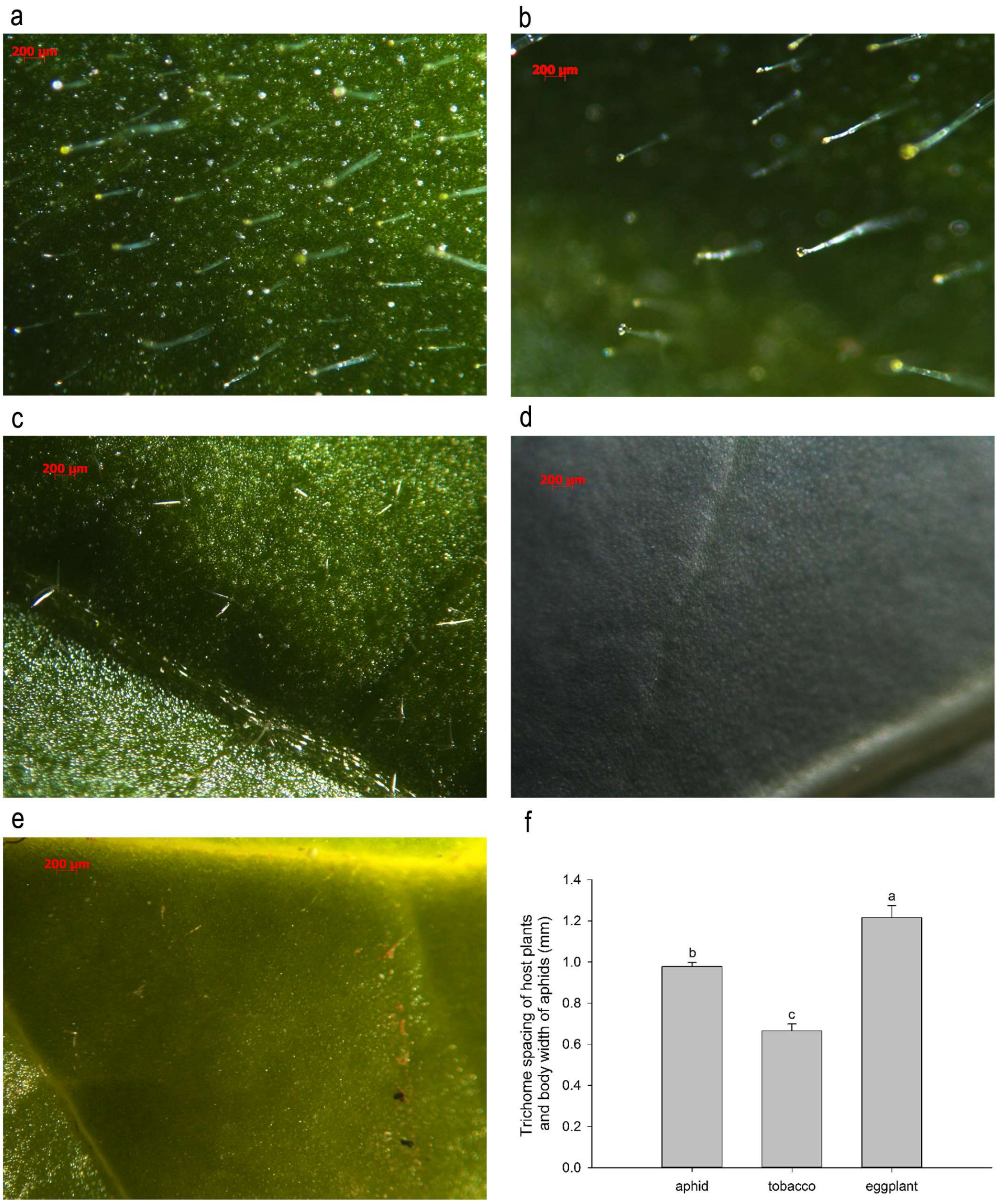
a
200 μm
b
200 μm
c
200 μm
d
200 μm
e
200 μm
f
Trichome spacing of host plants and body width of aphids (mm)
1.4
1.2
1.0
0.8
0.6
0.4
0.2
0.0
a
b
c
aphid
tobacco
eggplant

Figure S4 | Comparison between the relative abundance of *Buchnera aphidicola* and the proportion of essential amino acids in different plant species. Different letters indicate significant difference of symbiont abundance (lower case, light grey bar) and the proportion of essential amino acids (upper case, dark grey bar) ($P < 0.05$, Tukey's HSD test).

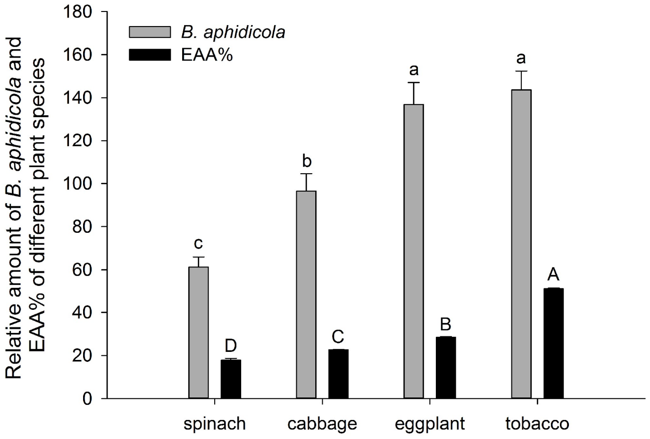

SUPPLEMENTARY TABLES

Table S1 | Amino acid concentration in four plant species.

Plant species	Replicate	EAAs (nM)	TAAs (nM)
cabbage	1	6857.54	30410.12
	2	6854.16	30310.59
	3	6799.44	30278.88
	4	6959.92	30454.85
	5	6901.30	30525.05
	mean	6874.47	30395.90
eggplant	1	3098.81	10484.86
	2	2958.01	10454.31
	3	2923.73	10425.76
	4	2876.37	10040.74
	5	2990.50	10903.68
	mean	2969.48	10461.87
tobacco	1	6434.73	12628.24
	2	6286.16	12349.49
	3	6230.13	12443.09
	4	6389.68	12196.49
	5	6286.64	12256.73
	mean	6325.47	12374.81
spinach	1	9803.30	45932.29
	2	10127.92	62515.47
	3	10407.07	60548.52
	4	10306.82	59643.44
	5	10345.35	61470.60
	mean	10198.09	58022.06

Table S2 | Performance (means ± SE) of *Myzus persicae* on different plant species estimated by using bootstrap techniques. The intrinsic rate of increase (*r*) calculated by TWOSEX-MSChart was used for measuring insect performance on different plant species.

Parameter	Plant species			
	cabbage	eggplant	tobacco	spinach
r (d^{-1})	0.1633 ± 0.0112 b	0.3143 ± 0.0107 a	0.1111 ± 0.0004 c	0.1417 ± 0.0157 bc

Table S3 | Precursor and product ions for liquid chromatography-mass spectrometry (LC-MS) analysis of 20 underivatized protein amino acids with their optimized values for collision energy.

Amino acid	Precursor ion [M+H]+(*m/z*)	Product ion (*m/z*)	Collision energy (eV)
ALA	90	44	20
ARG	175	116	35
ASN	133	87	35
ASP	134	74	35
CYS	122	76	35
GLN	147	130	35
GLU	148	130	35
GLY	76	30	20
HIS	156	110	35
ILE	132	86	35
LEU	132	86	35
LYS	147	130	35
MET	150	133	35
PHE	166	120	35
PRO	116	70	35
SER	106	60	35
THR	120	74	35
TRP	205	188	35
TYR	182	136	35
VAL	118	72	35

Table S4 | Primers used in this study.

Use	Organism	Target gene	Primer sequence (5' to 3')	Product size (bp)	Ref.
diagnostic PCR	*Buchnera aphidicola*	16S rRNA	Buch16S1F: GAGCTTGCTCTCTTTGTCGGCAA<br>Buch16S1R: CTTCTGCGGGTAACGTCACGAA	430	2
	Serratia symbiotica	16S rRNA	16SA1: AGAGTTTGATCMTGGCTCAG<br>PASScmp: GCAATGTCTTATTAACACAT	480	2
	Serratia symbiotica	groEL	GroEAF1: CCTCAAGGCTGTGGCCG<br>GroEAR1: TAGGCACCATCTCTGCAAACTC	150	2
	Regiella insecticola	16S rRNA	U99F: ATCGGGGAGTAGCTTGCTAC<br>16SB4: CTAGAGATCGTCGCCTAGGTA	200	2
	Hamiltonella defensa	16S rRNA	PABSF: AGCACAGTTTACTGAGTTCA<br>16SB1: TACGGYTACCTTGTTACGACTT	1660	2
	Rickettsia	16S rRNA	16SA1: AGAGTTTGATCMTGGCTCAG<br>Rick16SR: CATCCATCAGCGATAAATCTTTC	200	2
	Rickettsia	Citrate synthase	CS1: GGGGGCCTGCTCACGGCGG<br>CS2: ATTGCAAAAAGTACAGTGAACA	360	2
	Rickettsiella	16S rRNA	RCL16S-211F: GGGCCTTGCGCTCTAGGT<br>RCL16S-470R: TGGGTACCGTCACAGTAA TCGA	260	3
	PAXS	16S rRNA	10F: AGTTTGATCATGGCTCAGATTG<br>X420R: GCAACACTCTTTGCATTGCT	410	4
	Spiroplasma	16S rRNA	16SA1: AGAGTTTGATCMTGGCTCAG<br>TKSSspR: TAGCCGTGGCTTTCTGGTAA	510	2
	Spiroplasma	dnaA	ApDnaAF1: ATTCTTCAGTAAAAATGCTTGGA<br>ApDnaAR1: ACACATTTACTTCATGCTATTGA	450	2
	Wolbachia	16S rRNA	16S Wspecf: CATACCTATTCGAAGGGATAG<br>16S Wspecr: AGCTTCGAGTGAAACCAATTC	438	5
	Wolbachia	gatB	gatB_F1: GAKTTAAAYCGYGCAGGBGTT<br>gatB_R1: TGGYAAYTCRGGYAAAGATGA	471	5
	Wolbachia	hcpA	hcpA_F1: GAAATARCAGTTGCTGCAAA<br>hcpA_R1: GAAAGTYRAGCAAGYTCTG	515	5

	Wolbachia	coxA	coxA_F1: TTGGRGCRATYAACTTTATAG<br>coxA_R1: CTAAAGACTTTKACRCCAGT	487	5
	Wolbachia	ftsZ	ftsZ_F1: ATYATGGARCATATAAARGATAG<br>ftsZ_R1: TCRAGYAATGGATTRGATAT	524	5
	Wolbachia	fbpA	FbpA_F1: GCTGCTCCRCTTGGYWTGAT<br>FbpA_R1: CCRCCAGARAAAAYYACTATTC	509	5
	Wolbachia	wsp	81F: TGGTCCAATAAGTGATGAAGAAAC<br>522R: ACCAGCTTTTGCTTGATA	440	5
	Wolbachia	wsp	136F: TGAAATTTTACCTCTTTTC<br>691R: AAAAATTAAACGCTACTCCA	550	5
	Arsenophonus	16S rRNA	16SA1: AGAGTTTGATCMTGGCTCAG<br>Ars16SR: TTAGCTCCGGAGGCCACAGT	960	2
universal primers PCR	S-symbionts	rRNA	10F: AGTTTGATCATGGCTCAGATTG<br>480R: CACGGTACTGGTTCACTATCGGTC	2000-2500	6
fast detection PCR	*Serratia symbiotica*	23S rRNA	23SR-typeF2: AGCGCCGGTAAGGTGATATG<br>23SR-typeR2: CCGCCCTACTCATCGAACTC	307	
qPCR*	*Myzus persicae*	EF1α	ApEF1α107F: CTGATTGTGCCGTGCTTATTG<br>ApEF1α246R: TATGGTGGTTCAGTAGAGTCC	160	7
	Buchnera aphidicola	16S rRNA	qBuch16SF1: CGCAACCCTTATCCCCTGTT<br>qBuch16SR1: AGGGCCATGATGACTTGACG	105	
	Serratia symbiotica	16S rRNA	q16SR-typeF2: GACCAAAGTGGGGGACCTTC<br>q16SR-typeR2: CACATTACCCTTTCTCCTCGCT	272	

*Standard curve parameters and amplicon sequences of qPCR: *Myzus persicae* EF1α (E = 101.2%, R^2 = 0.999; 5'-CTGATTGTGCCGTGCTTATTGTCGCTGCTGGTACTGGAGAATTCGAAGCTGGTATTTCTAAAAATGGACAAACCCGTGAACACGCTCTATTGGCCTTCACCTTGGGTGTGAAACAATTGATCGTTGGTGTGAACAAGATGGACTCTACTGAACCACCATA-3');

Buchnera aphidicola 16S rRNA (E = 103.9%; R^2 = 0.998; 5'-AGGGCCATGATGACTTGACGTCGTCCCCACCTTCCTCCGGTTTATAACCGGCAGTCTCCTCTGAGTTCCCGGCCGAACCGCTGGCAACAGGGGATAAGGGTTGCG-3');

Serratia symbiotica 16S rRNA (E = 100.9%; R^2 = 0.996;

5’-GACCAAAGTGGGGGACCTTCGGGCCTCACGCCATCAGATGTGCCCAGGTGGGATTA
GCTGGTAGGTGGGGTAACGGCTCACCTAGGCGACGATCCCTAGCTGGTCTGAGAGGAT
GACCAGCCACACTGGAACTGAGACACGGTCCAGACTCCTACGGGAGGCAGCAGTGGG
GAATATTGCACAATGGGCGCAAGCCTGATGCAGCCATGCCGCGTGTGTGAAGAAGGCC
TTCGGGTTGTAAAGCACTTTCAGCGAGGAGAAAGGGTAATGTG-3’).

Table S5 | Aphid accessions tested for facultative symbionts. Accession number in bold was from this study.

Subfamily	Tribe	Aphid species	Facultative accession	Clade	Ref.
Eriosomatinae	Fordini	*Smynthurodes betae*	AY136159	A	8
Lachninae	Cinarini	*Cinara tujafilina*	EU348323	A	9
Lachninae	Cinarini	*Cinara cupressi*	EU348321	A	9
Aphidinae	Macrosiphini	*Macrosiphoniella helichrysi*	AY136151	A	8
Aphidinae	Macrosiphini	*Uroleucon caligatum*	AF293624	A	6
Aphidinae	Aphidini	*Aphis craccivora*	AY822594	A	1
Aphidinae	Macrosiphini	*Acyrthosiphon pisum*	AF293617	A	1
Aphididae	Macrosiphini	*Myzus persicae*	**KM577347**		
Lachninae	Tramini	*Trama troglodytes*	FJ655482	A	10
Lachninae	Cinarini	*Cinara ponderosae*	FJ655521	A	1
Lachninae	Cinarini	*Cinara terminalis*	FJ655485	A	1
Lachninae	Lachnini	*Lachnus takahashii*	FJ655522	A	1
Lachninae	Lachnini	*Lachnus shiicola*	FJ655544	A	1
Lachninae	Lachnini	*Lachnus tropicalis*	FJ655545	A	1
Lachninae	Lachnini	*Stomaphis pini*	FJ655524	A	1
Lachninae	Lachnini	*Stomaphis* sp.	FJ655523	A	1
Chaitophorinae	Chaitophorini	*Periphyllus bulgaricus*	AY136157	A	8
Lachninae	Cinarini	*Cinara whatolca*	FJ655525	A	1
Lachninae	Cinarini	*Cinara etsuhoe*	FJ655526	A	1
Lachninae	Lachnini	*Pterochloroides*	FJ655530	B	1
Lachninae	Lachnini	*Pterochloroides persicae*	AY136155	B	8
Lachninae	Cinarini	*Cinara cedri*	EU348324	B	9
Lachninae	Cinarini	*Cinara brauni*	FJ655519	B	1
Lachninae	Cinarini	*Cinara gudaris*	EU348317	B	9
Lachninae	Cinarini	*Cinara pinea*	EU348316	B	9
Lachninae	Cinarini	*Cinara pinea* group	FJ655520	B	1
Lachninae	Cinarini	*Cinara schimitscheki*	EU348318	B	9
Lachninae	Lachnini	*Lachnus roboris*	EU348314	B	9
Lachninae	Lachnini	*Tuberolachnus salignus*	EU348315	B	9
Lachninae	Lachnini	*Cinara edulis*	FJ655518	B	1
Lachninae	Lachnini	*Cinara* sp.	FJ655517	B	1
Lachninae	Cinarini	*Cinara maghrebica*	EU348319	B	9
Lachninae	Cinarini	*Cinara pilicornis*	EU348320	B	9
		Escherichia coli	AB045731		9

Table S6 | Raw qPCR data on endosymbionts abundances. Every number was the average of three technical replicates.

Aphids	*C*t			Aphids	*C*t		
	EF1α	*Buchnera*	*Serratia*		*EF1α*	*Buchnera*	*Serratia*
Myzus-cabbage1	20.98	14.73	35.47	*Myzus*-eggplant1	23.74	16.63	34.19
Myzus-cabbage2	22.18	15.80	36.10	*Myzus*-eggplant2	22.79	15.05	32.54
Myzus-cabbage3	20.96	13.71	36.70	*Myzus*-eggplant3	21.89	15.14	34.65
Myzus-cabbage4	21.94	15.15	34.43	*Myzus*-eggplant4	23.24	16.13	34.60
Myzus-cabbage5	20.72	13.66	36.67	*Myzus*-eggplant5	21.51	16.03	34.49
Myzus-cabbage6	22.69	16.03	35.82	*Myzus*-eggplant6	23.53	16.02	33.76
Myzus-cabbage7	21.10	14.49	37.15	*Myzus*-eggplant7	23.01	16.11	34.55
Myzus-cabbage8	20.00	13.80	37.64	*Myzus*-eggplant8	22.73	15.92	31.68
Myzus-cabbage9	21.56	13.52	33.68	*Myzus*-eggplant9	23.01	15.69	34.56
Myzus-cabbage10	20.67	14.60	34.14	*Myzus*-eggplant10	21.60	15.89	33.57
Myzus-tobacco1	23.78	16.77	34.87	*Myzus*-spinach1	21.96	15.94	35.04
Myzus-tobacco2	23.85	16.56		*Myzus*-spinach2	21.79	15.62	36.01
Myzus-tobacco3	23.83	16.57	33.57	*Myzus*-spinach3	21.86	15.62	36.93
Myzus-tobacco4	24.03	16.46	34.93	*Myzus*-spinach4	21.39	16.31	36.75
Myzus-tobacco5	23.56	16.48	34.57	*Myzus*-spinach5	21.32	15.93	36.39
Myzus-tobacco6	23.80	16.41	36.43	*Myzus*-spinach6	21.86	15.96	36.63
Myzus-tobacco7	23.33	16.41	36.93	*Myzus*-spinach7	22.42	16.08	34.47
Myzus-tobacco8	23.00	15.90	35.98	*Myzus*-spinach8	21.85	15.90	35.66
Myzus-tobacco9	23.02	16.35	35.21	*Myzus*-spinach9	22.24	16.35	35.73
Myzus-tobacco10				*Myzus*-spinach10	22.55	16.28	35.91

Note: The default data were *C*t values which had more than 0.5 deviation among the three technical replicates.

REFERENCE CITED IN SUPPLEMENTARY MATERIALS

1. Burke, G. R., Normark, B. B., Favret, C. & Moran, N. A. Evolution and diversity of facultative symbionts from the aphid subfamily Lachninae. *Appl. Environ. Microbiol.* **75**, 5328-5335 (2009).
2. Tsuchida, T., Koga, R., Shibao, H., Matsumoto, T. & Fukatsu T. Diversity and geographic distribution of secondary endosymbiotic bacteria in natural populations of the pea aphid, *Acyrthosiphon pisum*. *Mol. Ecol.* **11**, 2123-2135 (2002).
3. Tsuchida, T. et al. Symbiotic bacterium modifies aphid body color. *Science* **330**, 1102-1104 (2010).
4. Ferrari, J., West, J. A., Via, S. & Godfray, H. C. Population genetic structure and secondary symbionts in host-associated populations of the pea aphid complex. *Evolution* **66**, 375-390 (2012).
5. Simões, P. M., Mialdea, G., Reiss, D., Sagot, M. F. & Charlat, S. *Wolbachia* detection: an assessment of standard PCR protocols. *Mol. Ecol. Resour.* **11**, 567-572 (2011).
6. Sandström, J. P., Russell, J. A., White, J. P. & Moran, N. A. Independent origins and horizontal transfer of bacterial symbionts of aphids. *Mol. Ecol.* **10**, 217-228 (2001).
7. Oliver, K. M., Russell, J. A., Moran, N. A. & Hunter, M. S. Facultative bacterial symbionts in aphids confer resistance to parasitic wasps. *Proc. Natl. Acad. Sci. USA* **100**, 1803-1807 (2003).
8. Russell, J. A. & Moran, N. A. Costs and benefits of symbiont infection in aphids: variation among symbionts and across temperatures. *Proc. Biol. Sci.* **273**, 603-610 (2006).
9. Lamelas, A. et al. Evolution of the secondary symbiont "Candidatus *Serratia symbiotica*" in aphid species of the subfamily Lachninae. *Appl. Environ. Microbiol.* **74**, 4236-4240 (2008).
10. Normark, B. B. Evolution in a putatively ancient asexual aphid lineage: recombination and rapid karyotype change. *Evolution* **53**, 1458-1569 (1999).
